# The Role of Artificial Intelligence in the Identification and Evaluation of Bone Fractures

**DOI:** 10.3390/bioengineering11040338

**Published:** 2024-03-29

**Authors:** Andrew Tieu, Ezriel Kroen, Yonaton Kadish, Zelong Liu, Nikhil Patel, Alexander Zhou, Alara Yilmaz, Stephanie Lee, Timothy Deyer

**Affiliations:** 1 BioMedical Engineering and Imaging Institute, Icahn School of Medicine at Mount Sinai, New York, NY 10029, USA; 2 New York Medical College, Valhalla, NY 10595, USA; 3 Horace Mann School, Bronx, NY 10471, USA; 4 East River Medical Imaging, New York, NY 10021, USA; 5 Department of Radiology, Cornell Medicine, New York, NY 10021, USA

**Keywords:** artificial intelligence, deep learning, bone fracture, fracture detection

## Abstract

Artificial intelligence (AI), particularly deep learning, has made enormous strides in medical imaging analysis. In the field of musculoskeletal radiology, deep-learning models are actively being developed for the identification and evaluation of bone fractures. These methods provide numerous benefits to radiologists such as increased diagnostic accuracy and efficiency while also achieving standalone performances comparable or superior to clinician readers. Various algorithms are already commercially available for integration into clinical workflows, with the potential to improve healthcare delivery and shape the future practice of radiology. In this systematic review, we explore the performance of current AI methods in the identification and evaluation of fractures, particularly those in the ankle, wrist, hip, and ribs. We also discuss current commercially available products for fracture detection and provide an overview of the current limitations of this technology and future directions of the field.

## 1. Introduction

About 9.4 million fractures occur each year in the US [1], with an estimated annual expenditure of USD 22 billion from just osteoporotic fractures [2]. Due to an aging population, this is only expected to increase, with a projected cost of over USD 95 billion by 2040 [3]. Missed fractures are the most common diagnostic errors in the interpretation of musculoskeletal radiographs, and are especially prevalent in emergency department settings, where they account for approximately 80% of all misdiagnoses [4,5], leading to significant consequences such as delays in treatment and increased long-term morbidity [6].

Errors in fracture diagnosis are partially attributed to growing demands for radiological imaging, including radiography, computed tomography (CT), and magnetic resonance imaging (MRI) [7], with radiologist workloads estimated to have increased by 52% between 2012 and 2019 [8]. In the field of musculoskeletal radiology, increased usage of radiography is further compounded by the increasing incidence of fractures over the past 20 years, leading to radiologist fatigue and susceptibility to diagnostic errors [9]. Additionally, while the majority of skeletal radiographs are interpreted by radiologists in hospital settings, these reads may be performed by trainees or clinicians without specific musculoskeletal training [4], or even by nonradiologists [9], further contributing to missed fractures [5,10].

Artificial intelligence (AI), particularly machine learning, may provide a solution to several issues in the field of medical imaging analysis. Machine learning boasts a wide array of potential applications, including automated generation of diagnoses, image segmentation, and disease prognosis. One of the most common machine learning approaches to medical imaging analysis is deep learning, which uses deep neural network structures inspired by the human brain to interpret complex datasets [11]. In particular, convolutional neural networks (CNNs) exhibit strong performance with image-based tasks by using convolutional filters to automatically learn and extract features for image understanding. Trained using large imaging datasets, often with tens of thousands of images, these models are able to improve the accuracy and efficiency of clinician reads for fracture detection [12,13,14], with many standalone models achieving performances at the level of experienced clinicians or even outperforming them [13,15,16]. Applications of deep learning for fracture detection have been explored for a wide array of anatomical locations across various imaging modalities [12,17,18,19], and continue to grow as new capabilities of AI are being developed.

Given this rapidly evolving landscape, we provide a broad overview of the current state of machine learning in the identification and evaluation of bone fractures, particularly in the ankle, wrist, hip, and ribs. Specifically, “identification” refers to model tasks such as binary classification of fracture vs. no fracture, as well as fracture detection and segmentation, while “evaluation” describes additional tasks such as outcome prediction. We also highlight current commercial products available for fracture detection and discuss current challenges in the field, as well as future directions of this technology. As the applications of AI in medical imaging continue to broaden, an understanding of this technology will be invaluable as it begins to shape the practice of diagnostic radiology.

## 2. Methods

**Figure 1:**
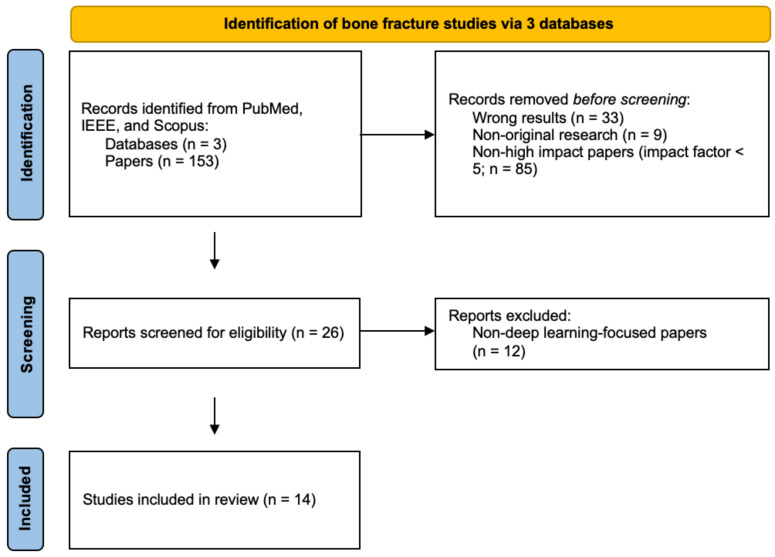
Flowchart of PRISMA study selection.

A systematic literature search was performed on three public journal databases (PubMed, IEEE, and Scopus) on 23 August 2023, as summarized in Figure 1. The PRISMA checklist [20] was used to facilitate transparent and complete reporting of the systematic review. Potential papers included those published since 1 January 2019, and were filtered using the following search terms: “ankle fracture” OR “wrist fracture” OR “hip fracture” OR “rib fracture” AND “artificial intelligence” OR “deep learning”.

All search records were first screened by title and abstract, and duplicate studies and data were excluded. Studies that did not use deep learning as their primary method of bone fracture detection were also excluded. From a total of 26 filtered papers during screening, the full texts were reviewed, and 14 studies were selected for discussion. Article selection was performed independently by two of the authors and collectively reviewed by the group before inclusion.

## 3. Performance Metrics

Given the large number of metrics used in evaluating the performance of imaging AI models, we provide here an overview of several commonly used metrics. Accuracy indicates the proportion of correctly classified cases relative to the total number of cases. Sensitivity, also known as recall, measures the proportion of true positives identified by the model among all ground truth positives, whereas specificity measures the ability of the model to identify true negatives among all ground truth negatives. Positive predictive value (PPV), also known as precision, complements sensitivity by indicating the proportion of true positives among all predicted positives by the model. Negative predictive value (NPV) similarly complements specificity, representing the proportion of true negatives among all predicted negatives by the model. The calculation of each of these metrics, bounded from 0 to 1, is shown below:

$$Sensitivity=\frac{TP}{TP+FN}$$

$$Specificity=\frac{TN}{TN+FP}$$

$$PPV=\frac{TP}{TP+FP}$$

$$NPV=\frac{TN}{TN+FN}$$

where TP is true positive, FP is false positive, TN is true negative, and FN is false negative.

Area Under the Curve (AUC) of the Receiver Operating Characteristic (ROC) curve provides a summary of the model’s overall performance, quantifying its ability to discriminate between positive and negative instances at all thresholds. An AUC score of 1 indicates the perfect performance of a model in discriminating between positive and negative instances, while a score of 0.5 indicates performance equivalent to random chance. For segmentation tasks, Dice score and intersection over union (IoU) are both commonly used metrics that evaluate the overlap between AI-predicted segmentations and the ground truth. For both metrics, a score of 1 indicates perfect overlap, while a score of 0 indicates no overlap at all.

$$Dice\;score=\frac{2\times Area\;of\;Intersection}{Total\;Area}$$

$$IoU=\frac{Area\;of\;Intersection}{Area\;of\;Union}$$

## 4. Ankle Fractures

**Figure 2:**
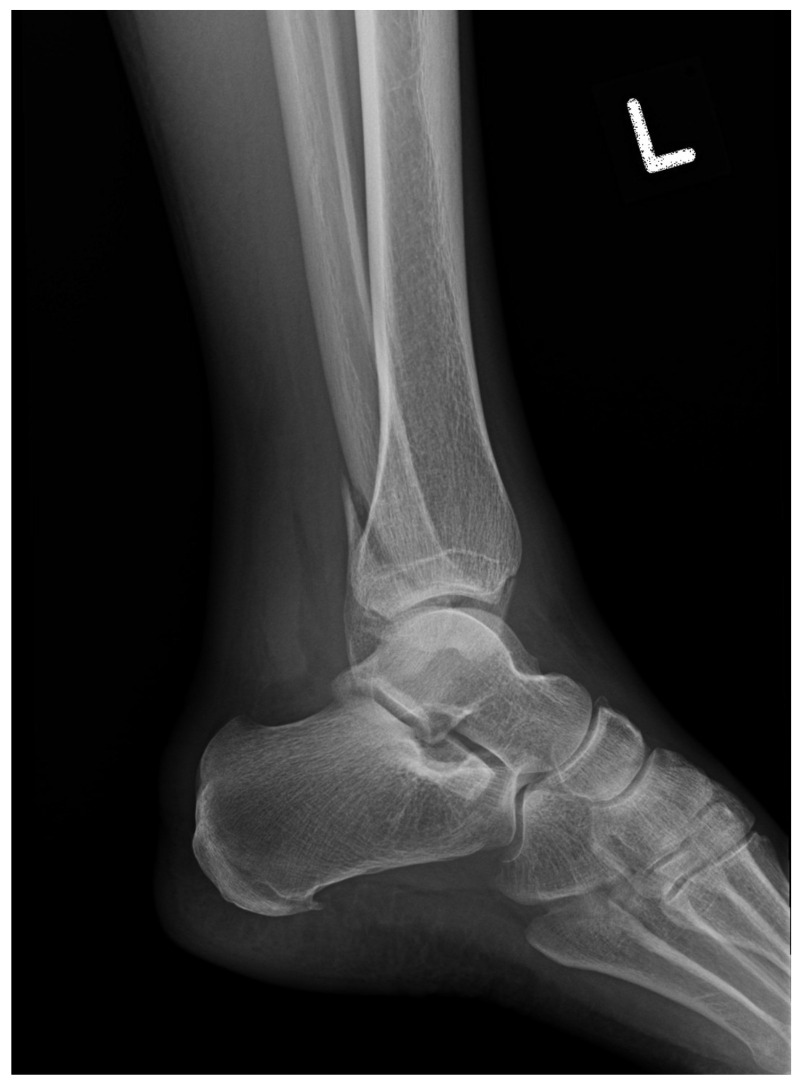
Distal fibular fracture as seen on lateral ankle radiograph.

Ankle fractures are among the most common injuries treated by orthopedic surgeons, accounting for 9% of all bone fractures with an estimated cost of USD 10 billion per year in the US [21]. The incidence of ankle fractures continues to increase, having tripled in elderly women over the past 30 years [22]. Treatment of ankle fractures relies on careful identification of bone lesions and damage to soft tissue and ligaments on both clinical assessment and imaging [23]. However, nearly 23% of ankle fractures are missed on initial radiographic imaging due to factors such as anatomical variance, superposition of structures on radiographs, lack of experience, and high physician workload [24,25]. Left untreated, these injuries can result in significant long-term morbidity [26,27]. An ankle fracture as seen on radiography is shown below in Figure 2.

Ashkani-Esfahani et al. sought to investigate the performance of deep-learning algorithms in the detection of ankle fractures, especially occult fractures [28]. A dataset of 1050 normal ankle radiographs was collected and matched with 1050 radiographs with ankle fractures, 72 of which were labeled as occult fractures due to being missed initially and subsequently diagnosed using additional radiographs or CT images. However, the group also hypothesized that incorporating multiple radiographic views would increase the detection of previously occult fractures. Using transfer learning, InceptionV3 [29] and Resnet-50 [30] models pre-trained with ImageNet [31] were trained with the ankle radiographs, with inputs as either single-view (AP) radiographs or 3-input structures with multiple views (AP, mortise, and lateral). Overall, the InceptionV3 model outperformed Resnet-50 in all performance criteria when using 3-view radiographic image stacks, including sensitivity (99% vs. 98%), specificity (99% vs. 94%), PPV (99% vs. 95%), NPV (99% vs. 97%), accuracy (99% vs. 96%), F1 score (99% vs. 96%), and AUC (99% vs. 98%). The incorporation of multiple views was important in achieving this performance, improving InceptionV3’s sensitivity (91% to 99%) and specificity (94% to 99%) when compared to the use of single views. Additionally, of the 72 occult fractures, the InceptionV3 model was able to detect 71/72 (98.6%) previously missed fractures, while Resnet-50 detected 69/72 (95.8%).

Given that previous work in ankle fracture detection was often bound by limitations such as large datasets [32], manual feature extraction [33], or pre-trained models [18], recent work by Kitamura et al. investigated whether comparable accuracy could be achieved by training CNNs de novo using a smaller dataset of 596 normal and abnormal ankle cases with multiple radiographic views [34]. The Inception V3, Resnet, and Xception [35] models were trained using single views, and ensembles were created from a combination of the trained models and then evaluated using three views for each ankle case. The ensembles achieved an accuracy of 81%, an impressive feat given the small dataset size and lack of reliance on manual feature extraction or pre-trained models.

The details and results of these studies are summarized in Table 1.

## 5. Wrist Fractures

**Figure 3:**
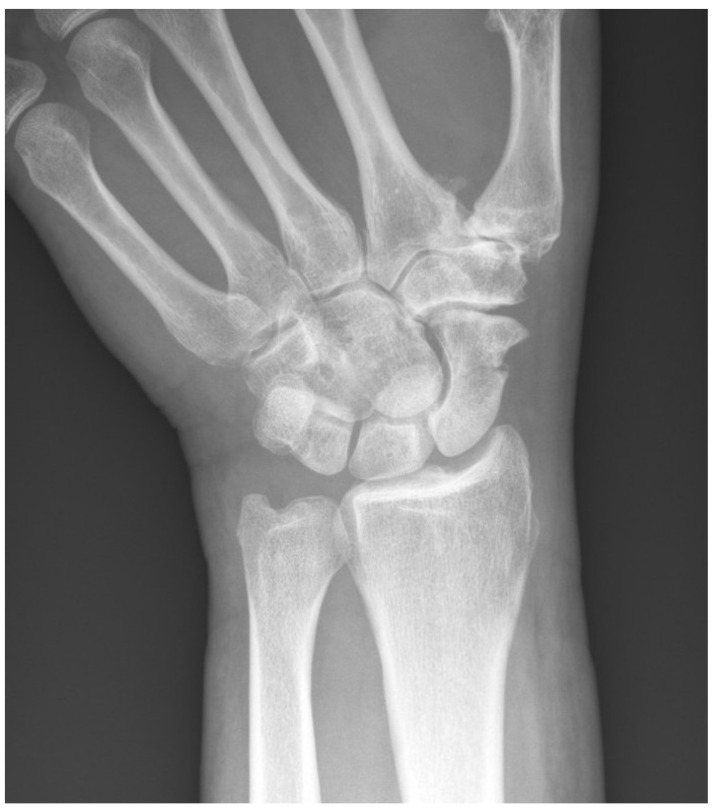
Scaphoid fracture as seen on ulnar-deviated PA scaphoid radiograph.

Fractures of the distal forearm and wrist are the most common sites of bone fracture in childhood, accounting for one-third of all cases [36]. Among the carpal bones, scaphoid fractures (as seen in Figure 3) occur most frequently, representing 2–7% of all skeletal fractures [37]. Left untreated, 12% of scaphoid fractures may progress to non-union [38], leading to further complications such as osteonecrosis, degenerative arthritis, and functional loss [39,40]. While radiography is generally the imaging modality of choice in evaluating such fractures, scaphoid fractures are often radiographically occult and difficult to diagnose, with estimations for occult fractures ranging from 7% to as high as 50% [41,42,43].

Given the challenges of scaphoid fracture detection, Langerhuizen et al. investigated the utility of deep-learning algorithms in the identification of such fractures, a field that has not yet been extensively explored [44]. A dataset comprised of 300 radiographic scaphoid series was assembled, consisting of 150 scaphoid fracture cases and 150 non-fracture cases, and used to train a convolutional neural network. On testing, the model achieved an AUC of 0.77 with 72% accuracy, 84% sensitivity, and 60% specificity. In comparison, a group of five orthopedic surgeons reading the images had similar accuracy (84%) and sensitivity (76%), but significantly increased specificity (93%; *p* < 0.01), owing to 13 false positive suggestions made by the model that were correctly identified by the clinicians. Interestingly, the model was able to detect 5 of 6 occult scaphoid fractures that were missed by all clinician readers.

A year later, Hendrix et al. expanded on these findings, developing a CNN capable of not only the detection of scaphoid fractures but also segmentation [14]. Given the small sample size of the previous study, a larger dataset was used, consisting of 1039 conventional radiographs of the hand, wrist, and scaphoid for training of the scaphoid segmentation CNN, as well as 3000 radiographs for scaphoid detection training. The segmentation model achieved a Dice score of 97.4%, while the fracture detection model achieved an AUC of 0.87 with 78% sensitivity, 84% specificity, and 83% PPV. This performance was similar to that of 11 clinician readers, who had an AUC of 0.83 (*p* = 0.09), thus achieving radiologist-level performance in the detection of scaphoid fractures.

Hendrix et al. continued this work the following year, using a further expanded dataset of 5004 conventional hand, wrist, and scaphoid radiographs for the training and testing of the scaphoid fracture detection model, this time also incorporating multi-view radiographs (PA, ulnar-deviated PA, oblique, lateral) [45]. The new model achieved a 72% sensitivity, 93% specificity, 81% PPV, and an AUC of 0.88. Although the AUCs for the model and that of five musculoskeletal radiologists (0.87) were again similar (*p* > 0.05), the reading time for four of the readers was significantly reduced with AI assistance (*p* < 0.001), with an average 51% reduction in reading time. Altogether, these three studies show that current state-of-the-art AI algorithms are able to match the performance of human clinicians in the detection of scaphoid fractures in conventional radiographs and improve diagnostic efficiency, but are yet to supersede the accuracy of human readers.

In addition to scaphoid fractures, AI methods have also been investigated in the detection of various other fractures of the wrist. In 2022, Hardalaç et al. evaluated the performances of several deep-learning models in detecting fractures of the radius and ulna using a collection of 542 pediatric wrist radiographs from 275 patients [46]. The models, which were pre-trained with the COCO dataset [47], included SABL [48], RegNet [49], RetinaNet [50], PAA [51], Libra R-CNN [52], FSAF [53], Faster R-CNN [54], Dynamic R-CNN [55], and DCN [56]. The best-performing individual model was the PAA model, achieving an AP50 of 0.754. Six ensemble models were then developed to further improve detection results, of which the WFD-C ensemble model displayed the strongest results with an AP50 of 0.864, an increase of 0.11 (14.59%) over the standalone PAA model.

Later work by Hržić et al. compared the performance of deep-learning models for wrist fracture detection to that of clinicians using a dataset of 19,700 pediatric wrist radiographs [13]. The exact types of wrist fractures included in the study and the distribution of these fractures were not specified. Several models based on YOLOv4 [57] and U-Net [58] architectures were trained, with the best-performing model (YOLO 512 Anchors) achieving an accuracy of 0.95 in binary fracture detection, 0.86 in appropriately counting the number of fractures, and 0.90 in fracture localization (defined as an IoU of greater than 0.5 between the predicted and true bounding boxes for each fracture). Compared to five radiologists, the YOLO 512 Anchors model performed better than four of the radiologists (*p* < 0.05) and at the same level as the fifth radiologist (*p* = 0.0654), with a model AUC of 0.965 compared to an average radiologist AUC of 0.831. When the radiologists utilized the model to assist their reads, their performances improved by an average F1-score of 0.067 (8.0%) with significant improvement in two of the readers (*p* < 0.05), leading the researchers to conclude that the model could feasibly be used to support clinical decision-making tasks in wrist fracture detection.

The details and results of these studies are summarized in Table 2.

## 6. Hip Fractures

**Figure 4:**
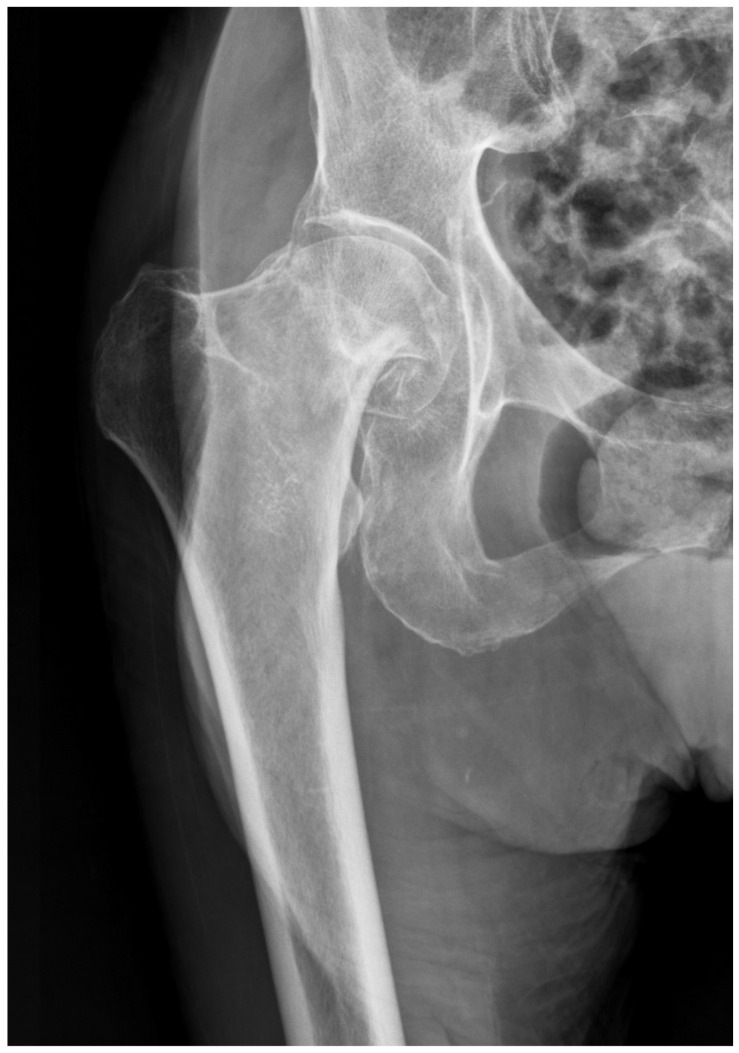
Femoral neck fracture as seen on pelvic radiograph.

Hip fractures are a common cause of hospitalization, morbidity, and mortality, accounting for the majority of fracture-related healthcare expenditure in men and women over 50 years old [61]. Among patients over 65, one in three will suffer a fall each year, with 10–15% of these falls resulting in a hip fracture [62]. While pelvic radiography is often used for the evaluation of these fractures (Figure 4), a minority of patients will have radiographically occult hip fractures, with reported rates between 3–10% among negative radiographs [63]. In such cases, additional imaging such as CT or MRI must be performed, resulting in increased diagnostic costs and delays in care [64]. AI may therefore provide a powerful tool in the diagnosis of previously occult hip fractures.

A systematic review and meta-analysis by Lex et al. evaluates the performance of AI in diagnosing hip fractures on pelvic radiographs as well as predicting postoperative outcomes such as mortality [65]. Of 39 included studies, 18 used AI for the diagnosis of hip fractures, while the other 21 focused on AI’s ability to predict postoperative outcomes, with a combined total of 754,537 radiographs used for the training, validation, and testing of machine learning models. On pooled data analysis, the odds ratio for the diagnostic error of the models as compared to clinician readers was calculated to be 0.79 (*p* = 0.36). For mortality predictions, the AUC of the models was 0.84, while traditional statistical methods using multivariable linear or logistic regression had an AUC of 0.79 (*p* = 0.09). As such, this study demonstrates that AI methods provide a promising approach for hip fracture diagnosis and prognosis from pelvic radiographs, although current implementations do not yet provide a substantial improvement over traditional methodologies.

Recent work by Kitamura continues to broaden the applications of AI in pelvic radiograph analysis, investigating the utility of deep learning not only for the detection of hip fractures, but also for pelvic fractures, acetabular fractures, radiograph positioning, and the presence of hardware [66]. A total of 14,374 radiographic images from 7440 patients were used to train and test the deep-learning models, with labels created to denote the presence or absence of hardware as well as radiograph positioning, which included pelvic, hip, and chest images, with each position further including a number of different views. For proximal femoral fracture detection, the model achieved an AUC of 0.95, which was comparable to other recent studies with AUCs of 0.97–0.99 [32,67] that required manual isolation of the femur using bounding boxes. Performances for fracture detection in other anatomical locations varied, with AUCs as low as 0.70 for posterior pelvic fractures and as high as 0.85 for acetabular fractures. For the detection of radiograph position and presence of hardware, the models achieved an AUC of 0.99–1.00. Altogether, this work highlights novel applications of deep learning in multiple aspects of pelvic radiography.

In order to more accurately omit false negatives during model development, Mawatari et al. developed a deep CNN for the detection of hip fractures in pelvic radiographs using CT and MRI as a gold standard [16]. The study used a dataset consisting of radiographs from a population of 316 patients with diagnosed proximal femoral fractures who had also received CT or MRI. The radiographs, which were manually annotated by radiologists with reference to CT and MRI for ROI selection, were then used to train the model, and the diagnostic performance of seven clinician readers with and without the CNN was then evaluated. The average AUC of the readers without the CNN was 0.832, which increased to 0.876 (*p* < 0.05) when guided by model output. Interestingly, the AUC of the CNN alone was 0.905, outperforming the combined readers even with CNN assistance. However, this was explained by variability in the experience levels of the readers, with the more experienced clinicians scoring higher than the CNN, achieving average AUCs of 0.934 and 0.920 with and without model interpretation, respectively.

The details and results of these studies are summarized in Table 3.

## 7. Rib Fractures

**Figure 5:**
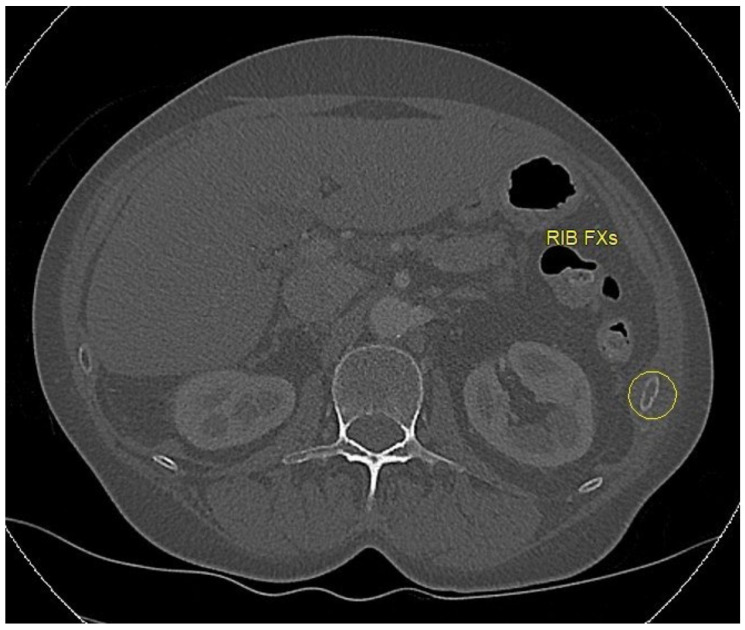
Left rib fracture (circled) as seen on axial CT.

Rib fractures are the most common injury in blunt chest trauma, with an estimated prevalence of 10–38% among all trauma patients [71]. The number and pattern of rib fractures are an important indicator of trauma severity, with an increased number of fractured ribs correlated with increased morbidity and mortality [72]. Unless identified and treated appropriately, rib fractures can present with life-threatening disease, particularly in elderly patients [73]. Among imaging modalities, plain radiography and CT are the most commonly used for rib fracture detection. Although radiography is fast and convenient, the detection rate is poor, missing over 50% of rib fractures [74]. On the other hand, while CT provides a more detailed assessment of rib fractures (Figure 5), it still presents with a misdiagnosis rate of 19.2–26.8% [75,76], and diagnosis can be tedious and difficult given the large amount of CT slices and the complex shape and course of the ribs across the numerous CT sections [77]. This presents a unique opportunity for machine learning to augment the accuracy and efficiency of rib fracture reads.

In 2020, Jin et al. developed the deep-learning model FracNet for the automatic detection and segmentation of rib fractures on CT images [78]. The model utilizes a dataset of 900 chest-abdomen CT scans with a total of 7473 annotated traumatic rib fractures. On testing, FracNet achieved a detection sensitivity of 92.9%, outperforming deep neural network counterparts such as 3D FCN [79] (87.8%) and 3D DeepLab [80] (91.3%) as well as reads from two expert radiologists (83.1%). FracNet also boasted an 86% decrease in reading time as compared to human clinicians, although it was associated with a higher number of false positives per scan (5.27 vs. R1: 1.34 and R2: 0.92 from the two readers, respectively). In the segmentation of rib fractures, FracNet continued to exhibit impressive performance, with a Dice score of 71.5% as compared to 3D FCN (66.2%), 3D DeepLab (68.7%), and clinician readers (64.7%).

Later work carried out by Zhang et al. in 2021 similarly used deep learning for rib fracture detection using a collection of CT images from 198 patients [81]. On testing, the trained model was able to identify 687 of 865 true fractures (79.4%), including a large number of fractures not originally detected by the two radiologists included in the study (R1: 75, R2: 66). When the clinicians utilized the model to augment their reads, they exhibited increased sensitivity in rib fracture detection (R1: 6.1%, R2: 4.8%; *p* < 0.05) as well as decreased reading times (R1: 36%, R2: 34%). Again, however, the standalone model had a higher false positive rate (0.43) than the two human readers (R1: 0.16, R2: 0.19; *p* < 0.001). Together, these studies show that deep-learning models may be a valuable asset in improving the sensitivity and efficiency of clinician rib fracture reads, although there are still challenges to using them as a standalone tool.

Recent work by Yao et al. further improved the precision of deep-learning methods with their Rib Fracture Detection System, a model utilizing a three-step algorithm for the detection of rib fractures in CT imaging consisting of bone segmentation, rib location, and rib fracture classification [82]. Using a dataset of annotated chest CTs from 1707 patients, their model achieved a precision of 0.869 and a recall of 0.913, outperforming competing algorithms such as FracNet, Fast RCNN [83], Faster RCNN, and YOLOv3 [84], and exhibiting comparable precision to that of two clinician readers (R1: 0.935, R2: 0.928) but with higher recall (R1: 0.693, R2: 0.853).

Given the focus of previous models on CT images, Gao et al. proposed a deep-learning method for automated rib fracture detection in digital radiographs [85]. CCE-Net, a novel network architecture for rib fracture detection based on the Faster RCNN framework, integrates contralateral, contextual, and edge-enhanced modules to improve AI detection of fractures. Using a dataset of 1639 radiographs with 2703 rib fractures, CCE-Net attained an AP50 of 0.911 and a recall of 0.934, an improvement of 15.76% and 6.74%, respectively, over the original Faster RCNN (0.787, 0.875). The model similarly outperformed other methods such as Libra RCNN (0.825, 0.886), Dynamic RCNN (0.887, 0.903), Cascade RCNN [86] (0.910, 0.929), and YOLO v4 (0.813, 0.881). The details and results of these studies are summarized in Table 4.

## 8. Commercial Availability

A number of AI products for bone fracture detection in these anatomical locations have already received clearance from the U.S. Food and Drug Administration (FDA). A comprehensive search for FDA-approved, commercially available products was conducted using the FDA webpage for AI and machine-learning-enabled medical devices [88], which is current as of 6 December 2023.

In May 2018, the FDA approved the marketing of the AI-based algorithm OsteoDetect (Imagen Technologies) for the detection of distal radial fractures in wrist radiographs [89]. The software localizes the fracture with a bounding box, achieving an AUC of 0.965, a sensitivity of 92.1%, and a specificity of 90.2%. Imagen Technologies also evaluated the performance of OsteoDetect in assisting 24 emergency medicine physicians with reading PA and lateral wrist radiographs, finding an improvement in overall AUC (0.84 to 0.89), sensitivity (75% to 80%), and specificity (89% to 91%).

Two years later, they also received clearance for their product FractureDetect, which similarly localizes bone fractures on radiographs but includes additional anatomical locations such as the shoulder, humerus, elbow, forearm, femur, knee, tibia, fibula, ankle, pelvis, hip, and clavicle [90]. The algorithm achieved an AUC of 0.98, a sensitivity of 95%, and a specificity of 89% on a testing dataset of 11,970 radiographs, and once again improved the performance of 24 clinician readers in overall AUC (0.91 to 0.95), sensitivity (82% to 90%), and specificity (89% to 92%).

uAI EasyTriage-Rib (Shanghai United Imaging Intelligence) received FDA clearance in 2021 as a workflow optimization algorithm that automatically detects and flags CT scans with three or more acute rib fractures, with an AUC of 0.94, a sensitivity of 93%, and a specificity of 85% [91]. Several months later, BriefCase for RibFx Triage (Aidoc Medical) received approval, an AI algorithm with similar functionality in notifying users of CT scans with three or more rib fractures [92]. FDA submission data showed an AUC of 0.976, a sensitivity of 96.7%, and a specificity of 90.4% in a multicenter study of 279 test cases. The company also showed that BriefCase had a time-to-notification of only 4.2 min, while the standard of care was associated with an 89.4 min delay between image acquisition and the time when a radiologist would first open the exam.

In 2022, BoneView (Gleamer) was approved, a software that provides bounding box localization of radiographic fractures in a variety of anatomical regions including the upper and lower extremities, pelvis, hip, shoulder, clavicle, ribs, and thoracic and lumbosacral spine, with a sensitivity of 93% and a specificity of 93% [93]. A study in 2021 funded by the company showed that BoneView enhanced the sensitivity (64.8% to 75.2%; *p* < 0.001) and specificity (90.6% to 95.6%; *p* = 0.001) of 24 clinician readers while reducing the overall reading time by 6.3 s per examination (*p* = 0.046) [94].

Later, that year saw the release of Rayvolve (AZmed), which offers similar functional capabilities as FractureDetect with additional fracture localization in the hands and feet [95]. The model’s standalone performance showed a sensitivity of 98.8%, a specificity of 88.6%, and an AUC of 0.986. Research conducted by the company also showed that usage of Rayvolve improved the diagnostic performance of 24 clinician readers, with an average increase in AUC from 0.846 to 0.893 (*p* = 0.0041), alongside increases in sensitivity (0.866 to 0.955) and specificity (0.826 to 0.831).

The performance of these products has also been externally validated by various studies. Recent work by Oppenheimer et al. demonstrated that implementation of BoneView led to increased diagnostic performance among residents in the study, with 25 additional fractures being identified in a dataset of 367 ground truth fractures, along with improvements in sensitivity (84.7% to 91.3%) and specificity (97.1% to 97.35%) [96]. Another study by Bousson et al. investigated the performance of various commercial algorithms, including Rayvolve and BoneView, in detecting acute fractures for patients admitted to the emergency department. The products demonstrated strong performance in daily radiological practice, with sensitivities of 92.6% and 91.3% and specificities of 70.4% and 90.5% for the two algorithms, respectively [97]. A summary table detailing the characteristics of these products is provided in Table 5.

## 9. Discussion

In summary, we detail recent contributions of AI methodology to the detection of bone fractures of the ankle, wrist, hip, and ribs, as well as currently available FDA-approved products for these tasks. However, there are countless additional applications of AI in musculoskeletal imaging. Numerous studies have investigated the performance of deep-learning methods for fracture detection in other anatomical locations such as the vertebrae [98,99], humerus [15], femur [32,100,101], shoulder [102,103], elbow [104,105], and skull [106]. While not fully covered within the scope of this review, we briefly summarize current AI methodologies for these types of fractures:

In the vertebrae, Shen et al., in 2023, trained a multitask detection network on 11,397 radiographic images, achieving an overall internal accuracy, sensitivity, and specificity of 97.41%, 84.08%, and 97.25%, respectively, and an overall external accuracy, sensitivity, and specificity of 96.85%, 83.35%, and 94.70%, respectively [98]. Zhang et al. in 2023 trained a U-Net/GCN/ResNet-based CNN model on 1217 CT images. For fracture detection, they achieved a sensitivity of 95.23%, an accuracy of 97.93%, and a specificity of 98.35%. For fracture classification, they achieved AUCs of 0.904, 0.945, 0.878, and 0.942 for the four types of vertebral fractures, respectively [99].

In the humerus, Chung et al., in 2018, trained a ResNet CNN on 1891 radiograph images of four types of humerus fractures, achieving an overall AUC of 0.996, sensitivity of 0.99, and specificity of 0.97 [15]. In the femur, Beyaz et al., in 2020, trained a CNN on 234 radiographic images with a genetic algorithm approach to optimize hyperparameters, achieving an accuracy of 79.3%, sensitivity of 82.9%, and specificity of 72.9% [100]. Similarly, Gale et al., in 2017, trained a DenseNet CNN on 53,278 radiographic images, achieving an AUC of 0.994, an accuracy of 97%, a precision of 99%, a recall of 95%, and an F1 score of 0.97 [32]. This was further validated by Oakden-Rayner et al. in 2022, who trained the same model on 45,786 radiographic images and achieved an AUC of 0.994 on internal validation and an AUC of 0.969 on external validation [101].

In the shoulder, Uysal et al., in 2021, trained 26 deep-learning models on the MURA X-ray dataset for two ensemble models, with the first achieving an accuracy of 0.846 and AUC of 0.886, and the second having an accuracy of 0.847 and AUC of 0.870 [102]. Magnéli et al. in 2023 trained a modified ResNet CNN on 7189 radiographic images, achieving an AUC of 0.96 for clavicle fractures and 0.87 for scapula fractures [103]. In the elbow, Rayan et al. in 2019 trained a multiview Xception CNN on 58,817 pediatric radiographic images, achieving an AUC of 0.95, accuracy of 88%, sensitivity of 91%, and specificity of 84% [104]. Luo et al., in 2021, took a knowledge-guided curriculum learning approach to train a multiview CNN model on 1964 radiographic images, achieving an AUC of 0.974 and an accuracy of 0.889 [105].

In the skull, Choi et al., in 2022, trained a YOLOv3 CNN on 413 radiographic images, achieving an AUC of 0.922, sensitivity of 81.1%, and specificity of 91.3% on an internal test set, and an AUC of 0.870, sensitivity of 78.9% and specificity of 88.2% on the external test set. Model-assisted AUC improvements of 0.094 and 0.069 were observed for radiology residents and emergency physicians, respectively, compared to diagnosis without AI assistance. However, no statistically significant improvement was observed in pediatric radiologists [106].

Expanding beyond bone fractures, models have also been developed for the diagnosis of ACL tears [107], meniscal tears [108,109], osteoarthritis [110], and cartilage lesions [111]. Other models are capable of automated grading of various musculoskeletal diseases [112,113,114] as well as augmenting several aspects of the image acquisition process, such as protocoling [115], reducing MRI acquisition times [116], and improving image quality [117]. These examples only touch the surface of what is possible with current technology, especially as the field continues to grow.

### 9.1. Limitations

While recent advances in machine learning have made applications of AI in bone fracture detection more feasible than ever, there remain numerous challenges to be addressed for both the development and implementation of AI methods. Conventional deep-learning methods, as used by the majority of studies in this review, require large amounts of annotated data, which is a tedious, time-consuming, and often prohibitively expensive process. Given the complex regulatory and privacy concerns associated with the sharing of medical images, the field currently suffers from an overall lack of high-quality annotated images, as many datasets are not publicly available for research purposes [118,119]. This issue can be seen in many of the studies covered by this review, which were generally performed as retrospective, single-center studies on internal datasets unless otherwise specified. Because images are acquired using different protocols varying by institution, this lack of external validation presents a major limitation to the generalizability and accuracy of developed models, as they may suffer from decreased performance when used on external datasets. Lack of standardization across datasets also leads to intrinsic model biases depending on the geographic locations, pathologies, and imaging modalities represented in a given dataset [120,121].

Similarly, imaging quality may also affect model performance in fracture detection. Work by Lu et al. proposes a reinforcement learning and transformer-based solution to assess and account for image distortion (e.g., motion artifacts, noise, contrast dosing) to improve diagnostic accuracy in coronary CT angiography [122]. While this has not yet been explicitly studied in the context of fracture detection to the best of the authors’ knowledge, techniques such as this may have utility in this space.

These issues generate questions surrounding the performance of proposed AI models in actual clinical settings. As shown in this review, AI methods are not devoid of making errors, with the inherent architecture of these models making it difficult to decipher the decision-making and rationale behind incorrect outputs. Due to the diversity of visual characteristics associated with any given pathology, even overall well-performing models may consistently misdiagnose specific subsets of cases, especially when imaging features are subtle or underrepresented [123]. Given that models generally do not report uncertainty behind decisions, this may lead to model outputs that would be clearly erroneous to a human reader. Issues surrounding the accountability of such decisions and their impact on patient care must be thoroughly and explicitly addressed before these models can be seamlessly integrated into clinical workflows.

### 9.2. Future Directions

The future of AI in radiology is promising. Current models are capable of clinical tasks such as diagnosis, prognosis, classification, and segmentation, with performances that match human clinicians and only continue to improve.

An important contribution to the field has been the recent improvement of self-supervised and unsupervised learning methods, which are able to learn from large amounts of data without the need for manual annotations [124]. Models using these approaches have already achieved similar or even better performances than models trained with traditional supervised learning methods [125,126,127], giving rise to the idea of AI foundation models. While most current models are trained for specific applications with limited fluidity in adapting to new tasks, foundation models are able to be trained just once on large-scale datasets and subsequently fine-tuned to perform a wide array of downstream tasks, offering newfound flexibility and generalizability. Current medical imaging foundation models are capable of tasks such as the automatic segmentation of medical images [128,129], cardiac function assessment with text report generation from echocardiograms [130], and the diagnosis and prognosis of ocular diseases associated with retinal images [131].

The question of how these algorithms will be implemented into clinical workflows remains unanswered. Numerous studies have already demonstrated the feasibility of AI in improving the accuracy and efficiency of clinician readers in addition to boasting impressive standalone performance. Potential applications of AI models in radiology include augmentation of reads, validation of results, or perhaps the offloading of simple diagnoses to AI to allow radiologists to focus on more cognitively challenging tasks, as suggested by Jha and Topol [132]. However, there is no easy answer to this question, as it involves a complex interplay between patients, radiologists, and other healthcare providers in addition to associated reimbursement policies and legal ramifications. We continue to await further research on how these algorithms will impact patient outcomes, which may help uncover the optimal way to utilize AI in clinical practice, especially as the scope of AI continues to evolve.

## 10. Conclusions

Deep-learning models for bone fracture detection in the ankle, wrist, hip, and ribs have achieved performance that is comparable or superior to that of clinician readers. These algorithms are already becoming commercially available for integration into clinical workflows, providing numerous benefits to radiologists such as increased diagnostic accuracy and efficiency. The utility of AI in fracture detection and radiology on a broader scale is an area of active research, and the capabilities of generated models continue to rapidly evolve. While their exact applications in clinical practice remain to be determined, AI methods have the potential to optimize radiologist workflows and enhance healthcare delivery for both patients and providers, and clinicians should be informed on the current state of AI as it will likely impact the future practice of radiology.

## Figures and Tables

**Table 1:** Summary of study characteristics for ankle fracture detection models.

Lead Author	Year	Imaging Modality	Total Number of Images *	Models Used	Model Tasks	Performance Metrics
Ashkani-Esfahani	2022	X-ray	6300	InceptionV3 Resnet-50	Fracture detection	InceptionV3: sensitivity 99%, specificity 99%, PPV 99%, NPV 99%, accuracy 99%, F1 score 99%, AUC 99% Resnet-50: sensitivity 98%, specificity 94%, PPV 95%, NPV 97%, accuracy 96%, F1 score 96%, AUC 98%.
Kitamura	2019	X-ray	1681	InceptionV3 Resnet-101 Xception	Fracture detection	Accuracy 81%, sensitivity 80%, specificity 83%, PPV 82%, NPV 81%

* Reported as the combined total of training, validation, and testing datasets.

**Table 2:** Summary of study characteristics for wrist fracture detection models.

Lead Author	Year	Imaging Modality	Region	Total Number of Images	Models Used	Model Tasks	Performance Metrics
Langerhuizen	2020	X-ray	Scaphoid	300	Pre-trained CNN (Visual Geometry Group) [59]	Fracture detection	AUC 0.77, accuracy 72%, sensitivity 84%, specificity 60%
Hendrix	2021	X-ray	Scaphoid	4229	DenseNet-121 [60]	Fracture detection, scaphoid segmentation	AUC 0.87, sensitivity 78%, specificity 84%, PPV 83%, Dice score 97.4%
Hendrix	2023	X-ray	Scaphoid	19,111	InceptionV3	Fracture detection and localization, scaphoid localization, laterality classification	AUC 0.88, sensitivity 72%, specificity 93%, PPV 81%
Hardalaç	2022	X-ray	Radius, Ulna (Pediatric)	542	SABL, RegNet, RetinaNet, PAA, Libra R-CNN, FSAF, Faster R-CNN, Dynamic R-CNN, DCN	Fracture detection and localization	AP50 0.864
Hržić	2022	X-ray	Wrist (Pediatric)	19,700	YOLOv4	Fracture detection, enumeration, and localization	AUC 0.965, accuracy 95%, sensitivity 95%, PPV 96%, F1 score 0.95 Fracture enumeration: Accuracy 86% Fracture localization: Accuracy 90%

**Table 3:** Summary of study characteristics for hip fracture detection models.

Lead Author	Year	Imaging Modality	Region	Total Number of Images	Models Used	Model Tasks	Performance Metrics
Lex	2023	X-ray	Femoral Neck Intertrochanteric Subtrocanteric	754,537 ^1^	Various models, including: AlexNet [68], GoogLeNet [69], ResNet-50, DenseNet-121, ResNet-18, PelviXNet [70], Faster RCNN	Fracture detection, outcome prediction	Diagnosis: odds ratio 0.79, sensitivity 89.3%, specificity 87.5%, F1 score 0.90 Postop mortality: AUC 0.84
Kitamura	2020	X-ray	Pelvic Acetabular Hip	14,374	DenseNet-121	Fracture detection, hardware detection, imaging position	Proximal femoral: AUC 0.95 Acetabular: AUC 0.85 Anterior pelvic: AUC 0.77 Posterior pelvic: AUC 0.70 Radiograph position: AUC 0.99 Hardware presence: AUC 1.00
Mawatari	2020	X-ray	Proximal Femoral	352	GoogLeNet	Fracture detection	AUC 0.905

^1^ Pooled total, with 39,598 images used for fracture detection and 714,939 for outcome prediction.

**Table 4:** Summary of study characteristics for wrist fracture detection models.

Lead Author	Year	Imaging Modality	Total Number of Images ^1^	Models Used	Model Tasks	Performance Metrics
Jin	2020	CT	900	FracNet	Fracture detection and segmentation	Sensitivity 93%, Dice score 71.5%
Zhang	2021	CT	198	Foveal Network [87] Faster R-CNN	Rib segmentation, fracture detection	Sensitivity 79.4%
Yao	2021	CT	1707	U-Net 3D DenseNet	Bone segmentation, fracture detection	Sensitivity 91%, PPV 87%, NPV 97%, F1 score 0.890
Gao	2022	X-ray	1639	CCE-Net	Fracture detection and localization	Sensitivity 93%, AP50 0.911

^1^ For CT, this is listed as total number of CT scans.

**Table 5:** Summary of FDA-approved AI products for bone fracture detection.

Product (Company)	Approval Year	Imaging Modality	Region	Functionality	Performance Metrics
OsteoDetect (Imagen Technologies)	2018	X-ray	Distal radius	Fracture detection and localization	AUC 0.97, sensitivity 92%, specificity 90%
FractureDetect (Imagen Technologies)	2020	X-ray	Ankle, clavicle, elbow, femur, forearm, hip, humerus, knee, pelvis, shoulder, tibia, fibula, wrist	Fracture detection and localization	AUC 0.98, sensitivity 95%, specificity 89%
uAI EasyTriage-Rib (Shanghai United Imaging Alliance)	2021	CT	Ribs	Notification if ≥3 fractures	AUC 0.94, sensitivity 93%, specificity 85%
BriefCase (RibFx) (Aidoc Medical)	2021	CT	Ribs	Notification if ≥3 fractures	AUC 0.98, sensitivity 97%, specificity 90%
BoneView (Gleamer)	2022	X-ray	Ankle, foot, knee, tibia, fibula, wrist, hand, elbow, forearm, humerus, shoulder, clavicle, pelvis, hip, femur, ribs, thoracic spine, lumbosacral spine	Fracture detection and localization	AUC 0.93, sensitivity 93%, specificity 93%
Rayvolve (AZmed)	2022	X-ray	Ankle, clavicle, elbow, forearm, hip, humerus, knee, pelvis, shoulder, tibia, fibula, wrist, hand, foot	Fracture detection and localization	AUC 0.99, sensitivity 99%, specificity 89%

## Data Availability

No new data were created or analyzed in this study. Data sharing is not applicable to this article.
